# The Role of Public Oral Health Services and Socioeconomic Factors in Oral Cancer Mortality in Brazil

**DOI:** 10.1111/jphd.12676

**Published:** 2025-04-06

**Authors:** José Mário Nunes da Silva, Maria Eduarda Macedo Vila‐Castro, Antônio Borges Nunes‐Neto, Fabrício Dos Santos Menezes

**Affiliations:** ^1^ Department of Statistics Federal University of Piauí Teresina Brazil; ^2^ Laboratório de Inferência Causal Em Epidemiologia (LINCE‐USP), School of Public Health University of São Paulo São Paulo Brazil; ^3^ Department of Dentistry Federal University of Piauí Teresina Brazil; ^4^ Department of Health Education Federal University of Sergipe Lagarto Brazil

**Keywords:** Brazil, mortality, oral cancer, oral health services, socioeconomic factors

## Abstract

**Objective:**

To assess the relationship between socioeconomic factors and the provision of oral health services in Primary Healthcare with mortality due to oral cancer in Brazil.

**Methods:**

This ecological study was conducted across 1105 Brazilian municipalities, encompassing 11,412 oral health teams (OHTs). The outcome variable was the oral cancer mortality rate, standardized by age and sex. Socioeconomic factors and variables related to the oral health services provided by municipal OHTs were considered explanatory variables. We employed multilevel Poisson regression models with random effects at the municipal level to assess the association between oral cancer mortality rates and explanatory variables.

**Results:**

At the municipal level, oral cancer mortality was negatively associated with higher human and social development and greater income inequality but positively linked to higher rates of population aging and greater coverage of primary and oral healthcare services. At the OHT level, mortality reduction was observed among those who received specialized support and had greater availability of consultations. Active case‐finding for cancerous lesions and care monitoring were also linked to lower mortality. Conversely, the identification of high‐risk vulnerable patients by these teams was associated with higher mortality.

**Conclusion:**

This study suggests that oral cancer mortality in Brazilian municipalities is influenced by socioeconomic factors and the availability of oral health services.

## 1 Introduction

Oral cancer is a significant cause of morbidity and mortality worldwide, representing a major public health concern [1]. In 2022, GLOBOCAN estimated 389,485 new cases of lip and oral cavity cancer and 188,230 deaths globally [2]. Brazil stands out in South America for having both the highest oral cancer incidence and mortality rates [1]. Between 1983 and 2017, there were 142,634 recorded deaths from oral and oropharyngeal cancers in the country, with 81% of these deaths occurring in men [3]. Furthermore, official estimates project around 15,100 new cases of oral cavity cancer annually for the 2023–2025 triennium, ranking it eighth among the most common nonmelanoma cancers [4].

The etiology of oral cancer is multifactorial, involving genetic, environmental, and behavioral factors [5]. Tobacco use and excessive alcohol consumption are the primary risk factors, particularly when combined [6]. Other predisposing factors include HPV infection, ultraviolet radiation exposure, a nutrient‐poor diet, inadequate oral hygiene, and a family history of oral cancer [5]. Evidence also suggests that individuals in socioeconomically disadvantaged situations are at greater risk of developing oral cancer and facing complications associated with the disease [1, 2, 7, 8, 9, 10].

In Brazil, Primary Healthcare (PHC) serves as the main entry point to the Unified Health System (SUS) [8]. PHC teams, composed of professionals from different fields, provide services and coordinate patient care across various settings and levels of the healthcare system [11]. Introduced in 2000, Oral Health Teams (OHTs) complement this model by offering basic dental care, health promotion, prevention, and referrals for more complex treatments [11]. Each OHT includes a dentist, dental assistant, and, in some cases, a dental technician, operating within specific territories through Basic Health Units (BHUs) [12]. Since the National Oral Health Policy (NOHP) was established in 2004, OHTs have played a crucial role in preventing and diagnosing diseases like oral cancer [13].

Despite the progress made since the introduction of the NOHP, including increased access to public oral health services, significant challenges remain in the organization and operation of OHTs. Key issues include inadequate infrastructure in BHUs, insufficient preventive activities, gaps in professional training and development, difficulties in integrating with the wider healthcare network [14, 15], and socioeconomic inequalities that hinder equitable access to services [7, 8].

In this context, previous research has explored the influence of social determinants and the availability of public health services on oral cancer mortality [7, 8, 9, 10, 16, 17]. However, few studies have specifically investigated the role of oral health services in this outcome. Thus, we hypothesize that the provision of oral health services, particularly those focused on preventive activities, combined with better socioeconomic conditions in municipalities, may be associated with reduced oral cancer mortality rates. Consequently, this study aims to assess the relationship between socioeconomic factors and the provision of oral health services in PHC with mortality due to oral cancer in Brazilian municipalities.

## 2 Methods

### 2.1 Study Design and Population

This is an observational ecological study with Brazilian municipalities as the unit of analysis. The study population comprised all Brazilian municipalities that reported at least three deaths due to oral cancer classified under codes C00 to C10 of the International Classification of Diseases—10th Revision (ICD‐10) during the period from 2016 to 2018, according to relevant literature [4, 8, 10, 18]. Additionally, we included only municipalities that had at least one OHT that was effectively evaluated in the third cycle of the National Program for Improving Primary Care Access and Quality (PMAQ‐AB).

### 2.2 Data Sources

We obtained data on oral cancer mortality from the Brazilian Mortality Information System (SIM), available through the Health Informatics Department (DATASUS) (http://www2.datasus.gov.br). SIM is a national epidemiological surveillance system responsible for collecting mortality data across Brazil, based on standardized death certificates nationwide. We also extracted population data for municipalities from DATASUS, using Intercensal Projections conducted by the Brazilian Institute of Geography and Statistics (IBGE), which were integrated into the database.

Data on oral health services provided by OHTs from the third cycle of the PMAQ‐AB were obtained from Module VI of the “External Evaluation Instrument for Oral Health Teams”, available at: https://www.gov.br/saude/pt‐br/composicao/saps/pmaq. This instrument was applied to 25,090 OHTs in 4974 Brazilian municipalities between July 2017 and August 2018. We specifically chose this cycle due to its high municipality participation rate and the significant increase in the number of questions related to oral cancer care compared to the previous cycle (from 3 to 10 questions).

Socioeconomic factors for municipalities were sourced from the Human Development Atlas in Brazil, provided by the United Nations Development Programme (http://www.atlasbrasil.org.br/acervo/biblioteca). Additional indicators were extracted from the 2010 Demographic Census provided by IBGE, available at: https://www.ibge.gov.br. Furthermore, we used the Primary Health Care Information System (e‐Gestor) (https://egestorab.saude.gov.br/) to extract information on primary and oral health care coverage during the study period.

### 2.3 Variables

The outcome variable of the study was the mortality rate, calculated as the ratio of the total number of oral cancer deaths to the estimated population of the municipality for the period from 2016 to 2018, expressed per 100,000 inhabitants. To enhance the robustness of our estimates, we included only municipalities with three or more recorded deaths and grouped the data into 3‐year intervals. This approach was adopted to minimize distortions in the analysis, caused by fluctuations in death reporting, particularly in smaller municipalities. Subsequently, the mortality rates were age‐ and sex‐standardized using the direct method, based on the Segi world standard population. It is noteworthy that deaths from municipalities with missing data were excluded from this analysis, accounting for 0.03% of cases.

At the municipal level, we analyzed the following contextual socioeconomic factors: Municipal Human Development Index (MHDI); life expectancy at birth; Gross Domestic Product (GDP) per capita; average household income per capita; social vulnerability index; Gini Index; Theil Index; rates of aging and unemployment; proportions of extreme poverty, and vulnerable to poverty. To facilitate international comparison, GDP per capita and average household income per capita were converted from Brazilian reais to US dollars, using the average exchange rates for 2017 (US$ 1.00 ≈ R$ 3.3) and 2010 (US$ 1.00 ≈ R$ 1.8), respectively. Additionally, we used the average coverage rates for primary and oral health care during the study period as measures of the quality and coverage of municipal health services (Table S1).

The variables related to the OHT level were selected based on their conceptual relevance in explaining oral cancer deaths. These variables were organized into the following categories: planning, monitoring, and evaluation of activities; matrix support; service organization; integration with healthcare networks; and specific actions targeting oral cancer care (Table S2).

### 2.4 Statistical Analysis

All variables were analyzed using descriptive statistics. After, a factor analysis was performed based on principal component analysis (PCA) due to the large number of municipal‐level variables and the significant correlations among related dimensions. The number of components to be extracted was determined using Kaiser's criterion (eigenvalue > 1) and Cattell's scree test. Additionally, we applied oblique rotation to enhance the interpretability of the factors, and the predicted scores of the extracted components were used in subsequent analyses.

We performed a Poisson regression analysis with robust variance to identify variables individually associated with the outcome, estimating the Incidence Rate Ratio (IRR) and the respective 95% confidence interval (95% CI). Variables with a *p*‐value < 0.10 were included in the multilevel analysis model.

**FIGURE 1:**
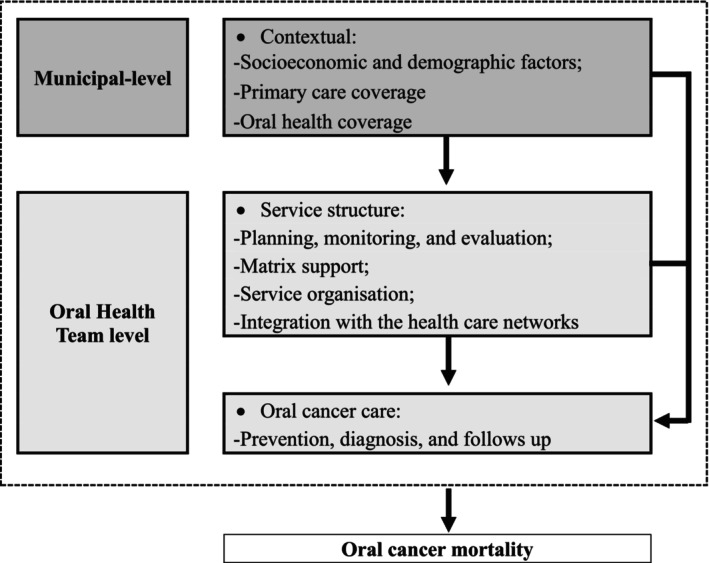
Hierarchical conceptual framework for factors associated with oral cancer mortality.

We conducted a two‐level multilevel mixed‐effects Poisson regression analysis to examine the influence of services provided by OHTs, as well as the socioeconomic context, on oral cancer mortality. The analysis initiated with a null model to identify significant random effects at the municipal level. Subsequently, variables were added to the null model in blocks according to their respective levels. Model 1 included only municipal‐level variables. Model 2 incorporated all significant variables from the previous model, along with oral OHT‐level variables with a *p*‐value < 0.10. Model 3 included all significant variables from the earlier models, as well as those specifically related to oral cancer care services (Figure 1). Changes in model fit were assessed using the likelihood ratio test. Finally, we examined the effect of each municipality on the outcome and generated a caterpillar plot, ranking the predicted values in ascending order along with their respective 95% CIs.

All analyses were conducted using Stata version 16.1 (StataCorp LP, College Station, Texas, USA) and R version 4.1.1 (R Core Team, Vienna, Austria) with the help of specific packages. The level of significance was set at *p* < 0.05. This study was reported in accordance with the RECORD guidelines.

## 3 Results

Between 2016 and 2018, the SIM recorded 18,834 deaths due to oral cancer across 3332 municipalities in Brazil. Out of the 4894 municipalities with at least one OHT evaluated in the 3rd cycle of the PMAQ‐AB, 1105 reported at least three deaths from oral cancer during the study period (Figure S1 and Figure S2). Consequently, the study encompassed 77.3% (*N* = 14,563) of the deaths during this period, with 38.1% occurring in the Southeast region, 31.5% in the Northeast, 19.4% in the South, 6.7% in the Central‐West, and 4.3% in the North.

The average standardized oral cancer mortality rate was 4.15 deaths per 100,000 inhabitants‐years. The lowest rates were observed in the municipalities of Guarapari, Espírito Santo (0.65/100,000 inhabitants‐years), and Nilópolis, Rio de Janeiro (0.68/100,000 inhabitants‐years), both located in the Southeast region. Conversely, the highest rates were recorded in Santa Amélia, Paraná (24.8/100,000 inhabitants‐years), and Turmalina, São Paulo (25.2/100,000 inhabitants‐years), situated in the South and Southeast regions, respectively (Figure S3).

A total of 11,412 OHTs were included in the study, with a median of six teams per municipality (IQR: 3–11). In 84 municipalities, only one team was evaluated, with 59.5% of these located in the Southeast region and 31% in the South. The three largest Brazilian capitals—Belo Horizonte, São Paulo, and Rio de Janeiro—had the highest number of evaluated OHTs, with 210, 262, and 307 teams, respectively (Figure S4).

Regarding contextual‐level variables, most Pearson correlation values were significant (Figure S5). The PCA identified three factors explaining 78.5% of the total variance (Figure S6). After being rotated by the Varimax method, eigenvalues were calculated and are presented in Table 1. The Kaiser–Meyer–Olkin (KMO) index was 0.819, indicating good fit of the sample, and Bartlett's test of sphericity was significant (*p* < 0.001). The first component, labeled as “human and social development”, accounted for 47.6% of the total variance. The second component, identified as “income inequality”, explained 19.5% of the total variance. Finally, the third component, named “aging and health coverage”, accounted for 11.4% of the total variance (Table 1). In the unadjusted municipal‐level analysis, all three components were associated with oral cancer mortality rates (Table 2).

**TABLE 1:** Rotated component matrix presenting factor loadings for the contextual variables included in the factor analysis at the municipal level.

Contextual variables	Component			Unexplained
	Human and social development	Income inequality	Aging and health coverage	
Municipal Human Development Index	−0.950			0.062
Average household income per capita ($)	−0.860			0.100
Gross Domestic Product per capita ($)	−0.640			0.570
Life Expectancy at Birth	−0.909			0.166
Social Vulnerability Index	0.889			0.135
Unemployment rate	0.533			0.448
% of extremely poor	0.878			0.172
% of vulnerable to poverty	0.978			0.033
Gini Index		0.972		0.052
Theil Index		0.977		0.038
Aging rate			0.737	0.403
Primary healthcare coverage			0.663	0.320
Oral health coverage			0.608	0.305
**% of variance explained**	**47.6**	**19.5**	**11.4**	—

*Note*: Rotation method was Varimax with Kaiser normalization.

**TABLE 2:** Descriptive analysis according to the levels and their association with oral cancer mortality rate.

Variables	*N*	%	IRR^a^	95% CI
Municipal‐level				
Human and social development				
−0.182 and more	576	52.1	Ref	
Up to −0.181 (better development)	529	47.9	0.90	0.85–0.96
Income inequality				
Up to −0.066	723	65.4	Ref	
−0.067 and more (more inequality)	382	34.6	0.88	0.82–0.94
Aging and Health coverage				
Up to 0.070	463	41.9	Ref	
0.071 and more (higher rates)	642	58.1	1.33	1.26–1.42
OHT‐level				
Planning, monitoring, and evaluation				
Investigates the epidemiological profile of the oral health population in the area^b^	6834	59.9	0.98	0.95–0.99
Conducts case discussions and therapeutic project planning^b^	6162	54.0	0.98	0.96–1.01
Matrix support				
Receives support from other professional teams	11,014	96.5	0.84	0.79–0.90
Receives support from Dental Specialty Centers	9287	81.4	0.82	0.79–0.84
Receives support from Family Health Support Centers	7451	65.3	0.97	0.95–0.99
Receives support from an oral and maxillofacial surgeon	9957	87.2	0.81	0.77–0.84
Receives support from periodontics specialist	9279	81.3	0.84	0.82–0.86
Receives support from oral medicine specialist	8555	75.0	0.89	0.87–0.92
Receives support from another specialist	4654	40.8	0.94	0.92–0.96
Service organization				
The OHT has received training to use the ICPC	10,847	95.0	0.94	0.89–0.98
Provides care for spontaneous demand	11,221	98.3	1.08	1.01–1.15
During user embracement, provides clinical care	11,205	98.2	1.09	1.03–1.14
The OHT identifies patient with higher risk of vulnerability	10,096	88.5	1.05	1.02–1.08
Integration with the health care network				
Provides emergency dental care outside of regular working hours	6936	60.8	0.87	0.84–0.88
Offers specialized consultations for referrals	10,822	94.8	0.78	0.74–0.83
Uses protocols for referring patients to other levels of care	10,612	93.0	0.90	0.86–0.94
Refers patients to specialists with information about the referral^b^	9798	85.9	0.93	0.91–0.96
Obtains feedback from specialists on referred patients with information about the service provided	9214	80.7	0.91	0.89–0.93
Oral cancer care				
Provides guidance on tobacco use	10,876	95.3	1.02	0.97–1.08
Provides guidance on alcohol and other drug use	10,704	93.8	1.02	0.97–1.06
Provides guidance on preventing exposure to solar radiation	10,607	89.1	1.02	0.99–1.05
Active case‐finding for cancerous lesions	9567	83.8	0.97	0.96–1.01
Systematically examines oral mucosa	10,412	92.3	1.01	0.96–1.07
Conducts other prevention and diagnostic activities	1.469	12.9	0.98	0.96–1.00
Performs biopsies at the basic health units	1060	9.3	1.12	1.08–1.15
Follows up and monitors the continuity of care	7955	69.7	0.95	0.92–0.98

*Note*: Municipal level; *n* = 1105; OHT level: *n* = 11,412.

Abbreviations: CI, confidence interval; ICPC, International Classification of Primary Care; IRR, incidence rate ratio; OHT, oral health team; Ref, reference.

^a^ Unadjusted Poisson regression model.

^b^ With documentation that proves it.

Table 2 also provides a description of the variables related to the services offered by the OHTs collected by PMAQ‐AB. A high proportion of OHTs (> 90%) were found to receive support from other professional teams, with service organization for training and care provision, specialist availability, and the use of protocols for patient referral within the healthcare network. Additionally, a considerable proportion of OHTs (> 80%) carried out activities specifically aimed at addressing oral cancer. These included providing guidance on tobacco use (95.3%), alcohol and drug use (93.8%), systematic examination of oral mucosa (92.3%), prevention of solar radiation exposure (89.1%), and active case‐finding for cancerous lesions (83.8%). The least implemented activity by OHTs was performing biopsies in BHUs (9.3%). In the unadjusted analysis, most variables initially showed associations with oral cancer mortality, particularly those related to matrix support, healthcare service organization, and coordination with the healthcare network (Table 2).

In the multilevel analyses, the initial null model indicated significant random effects across municipalities, with an initial variance of 0.262, which decreased to 0.232 (an 11.4% reduction) after the inclusion of contextual variables. In Model 1, better human and social development (IRR 0.85; 95% CI: 0.79–0.90) and higher inequality in income distribution (IRR 0.83; 95% CI: 0.78–0.89) were associated with reduced oral cancer mortality rates. In contrast, higher rates of aging and health service coverage (IRR 1.34; 95% CI: 1.27–1.42) were positively associated with the outcome. These effects remained consistent in subsequent models (Table 3).

**TABLE 3:** Multilevel mixed‐effect Poisson regression analysis of oral cancer mortality rates by municipal and oral health team levels.

Variables	Null model	Model 1	Model 2	Model 3
		IRR (95% CI)	IRR (95% CI)	IRR (95% CI)
Municipal‐level				
Human and social development (better development)		0.85 (0.79–0.90)	0.85 (0.80–0.91)	0.85 (0.80–0.91)
Income inequality (more inequality)		0.83 (0.78–0.89)	0.84 (0.79–0.89)	0.84 (0.79–0.89)
Aging and Health coverage (higher rates)		1.34 (1.27–1.42)	1.34 (1.26–1.42)	1.34 (1.26–1.42)
OHT‐level				
Receives support from Dental Specialty Centers			0.87 (0.85–0.89)	0.88 (0.84–0.90)
Receives support from Family Health Support Centers			0.94 (0.88–0.97)	0.95 (0.90–0.98)
Receives support from an oral and maxillofacial surgeon			0.89 (0.86–0.94)	0.91 (0.88–0.96)
Receives support from another specialist			0.95 (0.93–0.98)	0.96 (0.93–0.99)
Provides care for spontaneous demand			1.07 (0.99–1.13)	—
During user embracement, provides clinical care			1.08 (0.98–1.15)	—
The OHT identifies patient with higher risk of vulnerability			1.09 (1.04–1.13)	1.10 (1.02–1.14)
Offers specialized consultations for referrals			0.80 (0.76–0.88)	0.80 (0.73–0.92)
Obtains feedback from specialists on referred patients with information about the service provided			0.96 (0.94–0.99)	0.97 (0.93–1.00)
Active case‐finding for cancerous lesions				0.98 (0.96–0.99)
Performs biopsies at the basic health units				1.10 (1.03–1.19)
Follows up and monitors the continuity of care				0.97 (0.95–0.98)
Fixed effect				
Intercept (95% CI)^a^	3.53 (3.42–3.64)	3.42 (3.21–3.65)	3.52 (3.31–3.77)	3.60 (3.38–3.84)
Random effects				
Variance (SE)	0.262 (0.014)	0.232 (0.011)	0.209 (0.010)	0.208 (0.010)
LR test ($\chi^{2}$, *p*‐value)	1377.56 (< 0.001)	120.10 (*p* < 0.001)	108.08 (< 0.001)	94.32 (< 0.001)

*Note*: Model I: model adjusted for municipal‐level factors only; Model II: model adjusted for both municipal and oral health team level factors, maintaining the significance level of model I. Model III: model adjusted for both municipal and oral health team level factors, maintaining the significance level of model II.

Abbreviations: CI, confidence interval; IRR, incidence rate ratio; LR, likelihood ratio; OHT, oral health team; SE, standard error.

^a^ Values presented on a scale of 10^6^.

After incorporating variables at the OHT level, the final estimated variance decreased to 0.208 (a further reduction of 10.3%). In Model 2, support from Dental Specialty Centers (DSC), Family Health Support Centers (FHSC), oral and maxillofacial surgeons, and other specialists, along with the provision of specialized consultations and the receipt of referrals from other professionals within the healthcare network, was linked to reduced oral cancer mortality rates. Conversely, the identification of patients at higher risk of vulnerability by OHTs was associated with a 9% increase in the outcome (IRR 1.09; 95% CI: 1.04–1.13). These effects remained significant in most cases in Model 3 (Table 3).

In Model 3, active case‐finding for cancerous lesions and monitoring the continuity of care were associated with 2% (IRR 0.98; 95% CI: 0.96–0.99) and 3% (IRR 0.97; 95% CI: 0.95–0.98) reductions in oral cancer mortality rates, respectively. In contrast, performing biopsies in BHUs for cancer diagnosis was positively associated with the outcome (IRR 1.10; 95% CI: 1.03–1.19) (Table 3).

**FIGURE 2:**
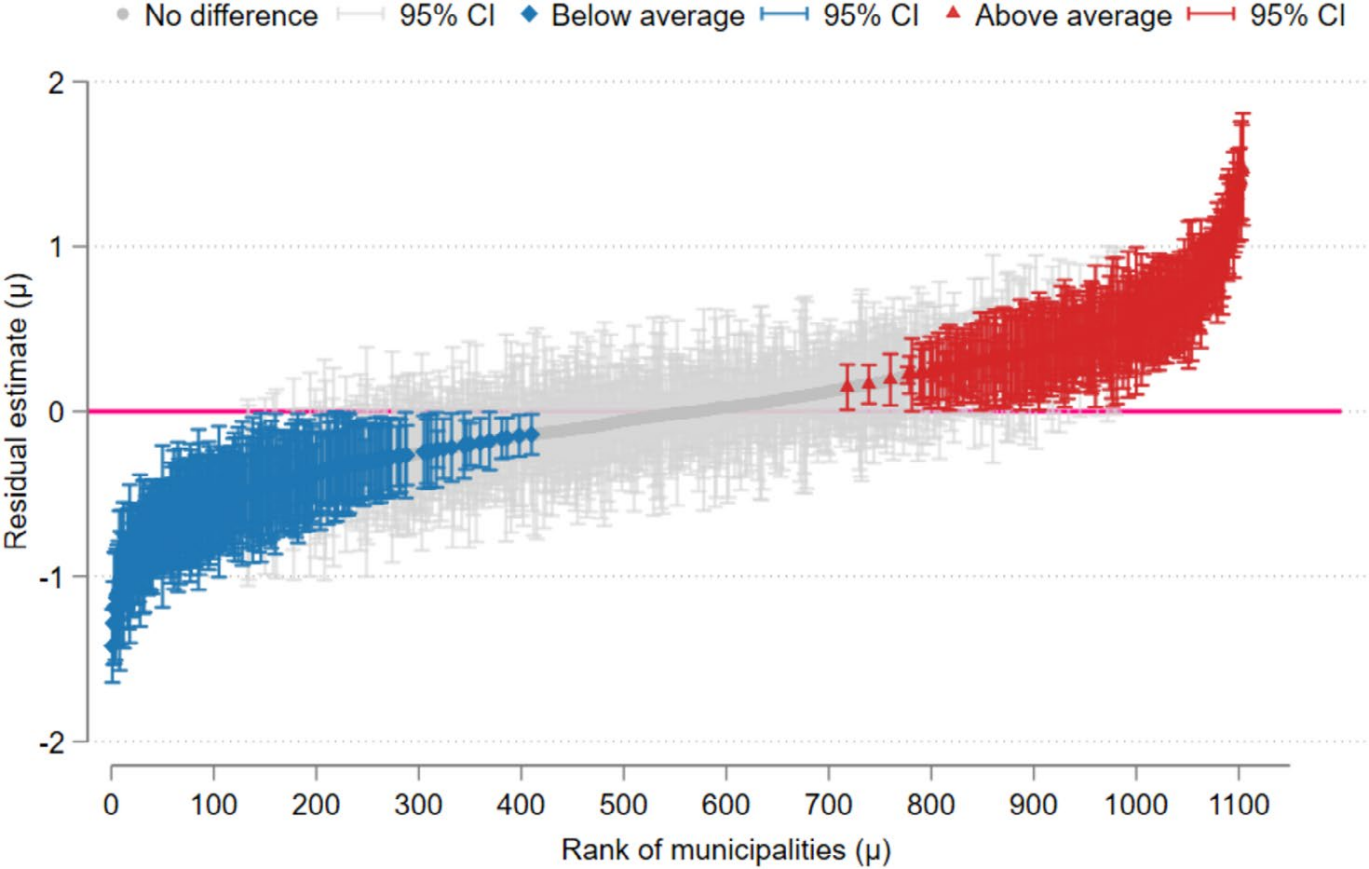
Caterpillar plot showing the effect of 1105 municipalities on oral cancer mortality and their respective 95% confidence intervals. [Color figure can be viewed at wileyonlinelibrary.com]

Finally, we used the predicted values from Model 3 to estimate the variability in oral cancer mortality rates across municipalities, as shown in Figure 2. In 258 municipalities (23.4%), the random effects and 95% CIs were below the overall average (zero line) and were considered to have better conditions. Conversely, in 268 municipalities (24.2%), the random effects and 95% CIs were above the general average (above the zero line), which we interpreted as suggesting worse conditions. For 52.4% of municipalities, it was not possible to distinguish them from the overall average due to overlapping 95% CIs with the zero line. The distribution of municipalities according to predicted values is presented in Figure S7.

## 4 Discussion

Our findings indicated that socioeconomic factors and the availability of primary oral health services within municipalities influence the oral cancer mortality rate in Brazil. Specifically, variables collected through the PMAQ‐AB, reflecting both the structure of oral health services and the specific actions taken by professionals to address oral cancer, were associated with reduced mortality in the municipalities analyzed. These results underscore the importance of oral health services not only in prevention but also in the early diagnosis and continuous monitoring of oral cancer cases, highlighting their impact on public health in Brazil.

We also identified a negative association between higher human and social development and oral cancer mortality rate, which aligns with previous literature on the topic [17, 19, 20, 21]. We hypothesize that municipalities with lower human and social development face greater exposure to risk factors and have limited access to cancer prevention and control services [5, 22]. This situation, combined with delayed diagnosis, may contribute to the higher oral cancer mortality rates [19]. Previous studies have explained this association by showing that oral cancer incidence is higher in developed regions, while mortality is greater in less developed areas, revealing disparities in access to early diagnosis and appropriate treatment [2, 17]. However, it is important to note that some studies have reported a positive relationship between human and social development, as represented by the HDI, and oral cancer mortality [8, 9, 23, 24], possibly due to underreporting or unmet demand [24].

Our analysis showed that income inequality was inversely related to the oral cancer mortality rate in municipalities. This finding is consistent with previous research [23] but contrasts with studies that found no significant association [8, 17, 18] or observed a positive relationship [9, 25]. These discrepancies may be explained by the fact that more disaggregated samples are often more effective at detecting associations, regardless of direction, which could clarify our results [9]. Additionally, we suggest that this inverse relationship may be linked to the fact that income inequality also reflects a lower life expectancy at birth, which does not favor longevity and may influence the lethality of chronic diseases such as cancer [23]. We also consider the possibility that underdiagnosis, due to a reduction in the number of OHTs in municipalities with greater income inequality between 2017 and 2019 [26], might partially explain this observed relationship, as suggested by a previous study [22].

We found that higher rates of population aging were associated with an increase in oral cancer mortality. This association aligns with the literature [8, 18, 24] that highlights how aging elevates cancer risk due to the accumulation of DNA damage, along with a decline in immune system function and the efficiency of cellular repair processes, increasing vulnerability to cancer [5, 27]. Moreover, prolonged exposure to environmental risk factors such as smoking and alcohol consumption, as well as hormonal changes associated with aging, also plays a crucial role in the development of the disease [6, 27].

In addition, a greater coverage of primary care and oral health services was linked to increased mortality due to oral cancer. We suggest that this association reflects the pro‐equity nature of PHC in Brazil, which prioritizes historically vulnerable regions that were previously underserved due to inadequate healthcare provision [19, 25]. In this context, the expansion of oral health services, driven by the NOHP, has been essential in addressing these past deficiencies and promoting access to care in communities that previously lacked adequate diagnosis and treatment [28]. This may have led to an increase in the detection of advanced cases of oral cancer, explaining the observed relationship between greater oral health coverage and deaths from this disease.

In this study, we observed that municipalities receiving support from DSC, FHSC, oral and maxillofacial surgeons, other specialists, provision of specialized consultations, and the receipt of referrals from other professionals within the healthcare network were associated with a reduction in oral cancer mortality. These initiatives not only enhance the performance of OHTs but also emphasize the fundamental importance of access to specialized oral health services in supporting the early detection and effective treatment of oral cancer [13, 21]. Studies have shown that a lack of access to specialized care is one of the primary causes of delayed diagnosis, reinforcing the need to optimize oral health services in PHC, particularly for vulnerable populations, to help reduce mortality from the disease [18, 19, 21]. Notably, the influence of matrix support is essential for the effective management of the disease and provides valuable insight into our findings.

In the scenario described earlier, DSCs play a vital role in complementing PHC by serving as referral units for the definitive diagnosis of suspected cases and for directing patients to appropriate oncological treatment [12, 29]. Our analysis found that approximately 90% of operational DSCs in the municipalities provided oral cancer diagnostic services. This is a significant finding, as the presence of referral units with qualified specialists is crucial for supporting and enhancing the work of OHTs [13], ensuring an integrated and effective approach to the early diagnosis and timely treatment of oral cancer. Previous literature partially supports our findings, indicating that a higher number of DSCs is associated with a reduction in hospitalized cancer cases, particularly among patients with advanced lesions [9, 30], and a consequent decrease in mortality [18].

The implementation of strategies such as active case‐finding for cancerous lesions and monitoring the continuity of care was associated with a reduction in oral cancer deaths in the municipalities studied. Given that these practices are outlined in key documents guiding primary oral health care in Brazil [12], our results are not surprising and contrast with a previous study [8]. In this context, the provision of these services highlights a better‐structured healthcare network, promoting more timely care, appropriate referrals, and effective case resolution. These practices strengthen the healthcare system's responsiveness, enabling quicker and more effective interventions, which are crucial for reducing oral cancer mortality [8, 13].

The observed association between performing biopsies in BHUs and oral cancer mortality may be explained by challenges in diagnosis and treatment. While offering biopsies in PHC enhances detection capacity, cases identified are often at advanced stages, leading to higher mortality rates. Furthermore, barriers to continuity of care, such as delays in referrals for specialized treatment and limitations in service quality, can compromise clinical outcomes [31]. This phenomenon may also reflect regional inequalities, as biopsies were the least implemented activity by the teams [29]. Thus, although performing biopsies represents progress, it must be accompanied by improved integration across levels of care and strategies to address inequalities in access [31], ensuring a meaningful impact on reducing mortality rates.

Despite these findings, the development of a public oral healthcare network within the SUS faces significant challenges, such as low primary care coverage, an inadequate number and unequal distribution of DSCs [26, 29]. Moreover, barriers like the lack of professional training and capacity for diagnosis and biopsy procedures, as well as unfavorable working conditions, persist [15, 31]. The reduction in health investment due to austerity policies and the recent impacts of the Covid‐19 pandemic also contribute to delayed diagnoses and, consequently, increased oral cancer mortality in Brazil [32].

### 4.1 Limitations and Strengths

This study has some limitations. Firstly, the use of secondary data may introduce measurement errors due to the possibility of inadequate or incomplete event records. However, it is important to note that death records in Brazil have significantly improved over the past two decades. The use of more recent data enhances the reliability of the information and helps mitigate potential limitations in the analysis of mortality data. Secondly, as an ecological study based on aggregated data, the results are applicable only at the population level and cannot be extrapolated to the individual level due to the risk of ecological fallacy. Additionally, the cross‐sectional nature of the data collected through PMAQ‐AB indicates that causal inferences must be made with caution. Third, to ensure greater stability in the data analysis, we included only municipalities with three or more oral cancer deaths. However, we recognize that this approach may not fully eliminate instabilities due to variations in population size and the complexity of data distributions. In addition, as PMAQ‐AB was a voluntary program, the availability of information across all municipalities was limited. Despite this, our study encompassed nearly 80% of the disease‐related deaths during the period and 50% of the OHTs evaluated by PMAQ‐AB, which strengthens confidence in our findings. Finally, we used socioeconomic factors only from the latest 2010 census. As a result, we were unable to assess recent socioeconomic trends and their relationship with oral cancer mortality rates. However, we believe that the socioeconomic differences between municipalities have remained proportional over the years, allowing us to consider the results obtained as robust.

Despite these limitations, our study integrated various databases representing different contexts in Brazil, enabling the extrapolation of results to similar settings. Furthermore, the standardization of rates, the use of various socioeconomic factors, and the analysis of different variables related to the service structure of oral health services—especially the actions aimed at oral cancer care derived from the largest Brazilian program for health service evaluation—add additional strength to the study.

## 5 Conclusion

This study suggests that oral cancer mortality in Brazilian municipalities is influenced by socioeconomic factors and the availability of oral health services. Lower mortality rates are observed in municipalities with higher human and social development, more income inequality, specialized support, and integrated healthcare focusing on prevention and early care. In contrast, municipalities with aging populations, broader primary healthcare coverage, and OHTs that identify highly vulnerable patients and perform biopsies in BHUs are associated with higher mortality rates. These findings underscore the need for increased investment in oral health policies that combine improvements and expansion in the services offered by primary care with enhanced socioeconomic conditions for more effective control of oral cancer in Brazil.

## Consent

This study utilized only publicly available secondary data in aggregate form, with no potential for individual identification. As such, it did not require informed consent or review by an Ethics Committee.

## Conflicts of Interest

The authors declare no conflicts of interest.

## Supporting information


**Data S1.** Supporting Information.

## Data Availability

The data that support the findings of this study are available in DATASUS at http://www2.datasus.gov.br. These data were derived from the following resources available in the public domain: ‐ Brazilian Mortality Information System, https://datasus.saude.gov.br/mortalidade‐desde‐1996‐pela‐cid‐10 Ministry of Health of Brazil, https://www.gov.br/saude/pt‐br/composicao/saps/pmaq Ministry of Health of Brazil, https://egestorab.saude.gov.br/paginas/acessoPublico/relatorios/relHistoricoCoberturaAB.xhtml Human Development Atlas in Brazil, http://www.atlasbrasil.org.br/acervo/biblioteca.
