# Supplementary material for: The Role of Public Oral Health Services and Socioeconomic Factors in Oral Cancer Mortality in Brazil
Source: J Public Health Dent. 2025 Apr 6;85(3):292–301. doi: 10.1111/jphd.12676 (PMC12418725; doi:10.1111/jphd.12676)
Supplement: Supplementary file 1 — Data S1. Supporting Information. [file JPHD-85-292-s001.docx]

**Supplemental Material**

*Journal of Public Health Dentistry*

**The role of public oral health services and socioeconomic factors in oral cancer mortality in Brazil**

José Mário Nunes da Silva MSc, PhD^1,2^, Maria Eduarda Macedo Vila-Castro^3^, Antônio Borges Nunes-Neto^3^, and Fabrício dos Santos Menezes DDS, PhD^4^

^1^ Department of Statistics, Federal University of Piauí, Teresina, Brazil

^2^ Laboratório de Inferência Causal em Epidemiologia (LINCE-USP), School of Public Health, University of São Paulo, São Paulo, Brazil

^3^ Department of Dentistry, Federal University of Piauí, Teresina, Brazil

^4^ Department of Health Education, Federal University of Sergipe, Lagarto, Brazil

**TABLE OF CONTENTS**

**TABLE S1 –** Description of the socioeconomic indicators used in the study…………………3

**TABLE S2** –Description of the variables related to the structuring of oral health services available in PMAQ-AB..……………………………………………………………………….5

**FIGURE S1 –** Flowchart of study population selection.……………………………………....7

**FIGURE S2** – Average number of deaths across the 1,105 Brazilian municipalities included in the study from 2016 to 2018……………………………………………………………………8

**FIGURE S3** – Age- and sex-standardized oral cancer mortality rate in Brazil from 2016 to 2018.……………………………………….………………………. ………………………….8

**FIGURE S4** –Distribution of the number of oral health teams in Brazilian municipalities participating in the PMAQ-AB included in the study…………………………………………..9

**FIGURE S5 –** Correlation matrix of contextual variables………………………………….…9

**FIGURE S6 –** Scree plot depicting the percentage of variance explained by each PCA component…………………………………………………………………………………….10

**FIGURE S7** – Effect of Brazilian municipalities on oral cancer mortality rates according to predicted values from the multilevel Poisson regression model………………………………11

**REFERENCES**………………………………………………………………………………11

**TABLE S1 –** Description of the socioeconomic indicators used in the study.

| **Category** | **I****ndicators** | **Description** | **Data source** |
| --- | --- | --- | --- |
| Human development | Municipal Human Development Index | Geometric mean of the indices for the dimensions of Income, Education, and Longevity, with equal weights. The scale ranges from 0 (worst) to 1 (best). | HDA [1] |
| Income | GDP per capita (US$) | Sum of municipal assets divided by the total municipal population as of 2017. | IBGE census [2] |
|  | Average household income per capita (US$) | Ratio of the total income of all individuals residing in permanent private households to the total number of these individuals. Values are in Brazilian reais (R$1.80), converted to US dollars ($1.00) as of August 1, 2010. | HDA [1] |
| Income distribution | Gini Index (%) | It measures the degree of income inequality within a given group, indicating the disparity between the incomes of the poorest and the richest. It is calculated as a ratio of the areas in the Lorenz curve diagram. | HDA [1] |
|  | Theil Index (%) | It measures the disparity between different groups within a population, taking into account both the inequality within each group and the inequality between groups. The index is calculated as the natural logarithm of the ratio between the arithmetic mean and the geometric mean of the average per capita household income. | IBGE census [2] |
|  | Social Vulnerability Index | Arithmetic means of the indices for the dimensions: urban infrastructure index, human capital index, and income and employment index. The scale ranges from 0 (best) to 1 (worst). | HDA [1] |
|  | % of extremely poor | Percentage of people with per capita household income equal to or below R$70.00 per month (US$40), in August 2010. | IBGE census [2] |
|  | % of vulnerable to poverty | Percentage of people with per capita household income equal to or below R$255.00 per month (US$145), in August 2010 reais, equivalent to half the minimum wage. | IBGE census [2] |
| Demographic | Geographic region | Grouping of federative units elaborated by IBGE, divided into five regions: Central-West (3 states and one federal district), Northeast (9 states), North (7 states), Southeast (4 states), and South (3 states). | IBGE census [2] |
| Employment | Unemployment rate | Percentage of the economically active population (EAP) in that age group who were unemployed, i.e., those who were not employed in the week prior to the census date but had been seeking work in the month leading up to the survey. | HDA [1] |
| Ageing | Ageing rate | Ratio of the population aged 65 or over to the total population, multiplied by 100 | HDA [1] |
| Health | Life expectancy at birth | Average number of years people are expected to live from birth, assuming that the level and pattern of age-specific mortality rates prevalent in the year of the census remain constant throughout their lifetime. | HDA [1] |
|  | Primary health care coverage (%) | Estimate of the population covered by primary care teams, multiplied by 100. | e-Gestor [3] |
|  | Oral health care coverage (%) | Estimate of the population covered by oral health teams, multiplied by 100. | e-Gestor [3] |

*Abbreviations:* GDP, gross domestic product; HDA, human development atlas in Brazil (Atlas do desenvolvimento humano do Brasil, in Portuguese); IBGE, Brazilian Institute of Geography and Statistics (Instituto Brasileiro de Geografia e Estatística, in Portuguese); e-Gestor, primary health care information system (Sistema de informação e gestão da atenção básica, in Portuguese).

**TABLE S2** – Description of the variables related to the structuring of oral health services available in PMAQ-AB [4].

| **Questions** | **Code in the database** | **Categories in the study** |
| --- | --- | --- |
| **2. Planning, monitoring, and evaluation (2/6 questions)** | VI.4 |  |
| 2.3 Did the OHT investigate the epidemiological profile of oral health in the population of the area?^a^ | VI.4.3.1 | 0 “No” 1 “Yes” |
| 2.4 Does the OHT conduct case discussions and therapeutic project planning?^a^ | VI.4.4.1 | 0 “No” 1 “Yes” |
| **3. Matrix Support (2/3 questions)** | VI.5 |  |
| 3.1 Does the OHT receive support from other professional teams to assist with the resolution of complex cases? | VI.5.1 | 0 “No” 1 “Yes” |
| 3.2.1 Does the OHT receive support from Dental Specialty Centers? | VI.5.2.1 | 0 “No” 1 “Yes” |
| 3.2.3 Does the OHT receive support from Family Health Support Centers? | VI.5.2.3 | 0 “No” 1 “Yes” |
| 3.3.1 Does the OHT receive support from an oral and maxillofacial surgeon | VI.5.3.1 | 0 “No” 1 “Yes” |
| 3.3.3 Does the OHT receive support from periodontics specialist? | VI.5.3.3 | 0 “No” 1 “Yes” |
| 3.3.4 Does the OHT receive support from oral medicine specialist? | VI.5.3.4 | 0 “No” 1 “Yes” |
| 3.3.9 Does the OHT receive support from another specialist? | VI.5.3.9 | 0 “No” 1 “Yes” |
| **4. Service organization (4/20 questions)** | VI.6 and VI.7 | 0 “No” 1 “Yes” |
| 4.2 Did the team receive training or qualification to use the International Classification for Primary Care? | VI.6.3 | 0 “No” 1 “Yes” |
| 4.5 Does the clinical care provided by the OHT ensure spontaneous demand? | VI.7.3.2 | 0 “No” 1 “Yes” |
| 4.6.1 Does the OHT provide clinical care during the user embracement? | VI.7.6.4 | 0 “No” 1 “Yes” |
| 4.9 Have the oral health professionals involved in user embracement been trained to assess and classify patient risk and vulnerability? | VI.7.10 | 0 “No” 1 “Yes” |
| **6. Integration with the health care network (5/6 questions)** | VI.10 |  |
| 6.1 Is there an emergency dental care service available in the health network outside of regular working hours? | VI.10.1 | 0 “No” 1 “Yes” |
| 6.2 Is there an offer for specialist consultations within the health network to which the dental health team can refer patients? | VI.10.2 | 0 “No” 1 “Yes” |
| 6.3 Does the OHT use protocols for referring patients to other levels of care within the health network? | VI.10.3 | 0 “No” 1 “Yes” |
| 6.4 Does the dentist refer patients to specialists with information about referral?^a^ | VI.10.4.1 | 0 “No” 1 “Yes” |
| 6.5 Does the oral health team receive feedback from specialists about referred patients with information about the service provided? | VI.10.5 | 0 “No” 1 “Yes” |
| **7. Oral cancer care (4/10 questions)** | VI.11 |  |
| 7.1 Does the OHT conduct oral cancer prevention and diagnostic activities? | VI.11.1 | 0 “No” 1 “Yes” |
| 7.2.1 Provide guidance on tobacco use? | VI.11.2.1 | 0 “No” 1 “Yes” |
| 7.2.2 Provide guidance on alcohol and other drug use? | VI.11.2.2 | 0 “No” 1 “Yes” |
| 7.2.3 Provides guidance on preventing exposure to solar radiation? | VI.11.2.3 | 0 “No” 1 “Yes” |
| 7.2.4 Active case-finding for cancerous lesions and community-based case detection? | VI.11.2.4 | 0 “No” 1 “Yes” |
| 7.2.5 Systematically examines oral mucosa? | VI.11.2.5 | 0 “No” 1 “Yes” |
| 7.2.6 Conduct other prevention and diagnostic activities? | VI.11.2.6 | 0 “No” 1 “Yes” |
| 7.3 Does the OHT perform biopsies at basic health units? | VI.11.3 | 0 “No” 1 “Yes” |
| 7.9 Does the OHT follow up and monitors the continuity of care? | VI.11.9 | 0 “No” 1 “Yes” |

*Abbreviations:* OHT, oral health team; PCT, primary health team; PMAQ-AB, National Program for Improving Primary Care Access and Quality (Programa Nacional de Melhoria do Acesso e da Qualidade da Atenção Básica, in Portuguese).

^a^ With documentation that proves it.

25,090 Oral Health Teams (OHTs) evaluated in the 3rd PMAQ-AB cycle (2017-2018) across 4,974 municipalities

22,993 OHTs successfully interviewed in 4,894 municipalities

18,834 recorded deaths from oral cancer (2016–2018) in 3,332 municipalities

17,002 recorded deaths from oral cancer in 2,922 municipalities, with 17,959 OHTs

A total of **14,563** deaths from oral cancer in **1,105** municipalities, with **11,412** OHTs included

1,817 municipalities with fewer than 3 deaths (N=2,439)

**FIGURE S1 –** Flowchart of study population selection.


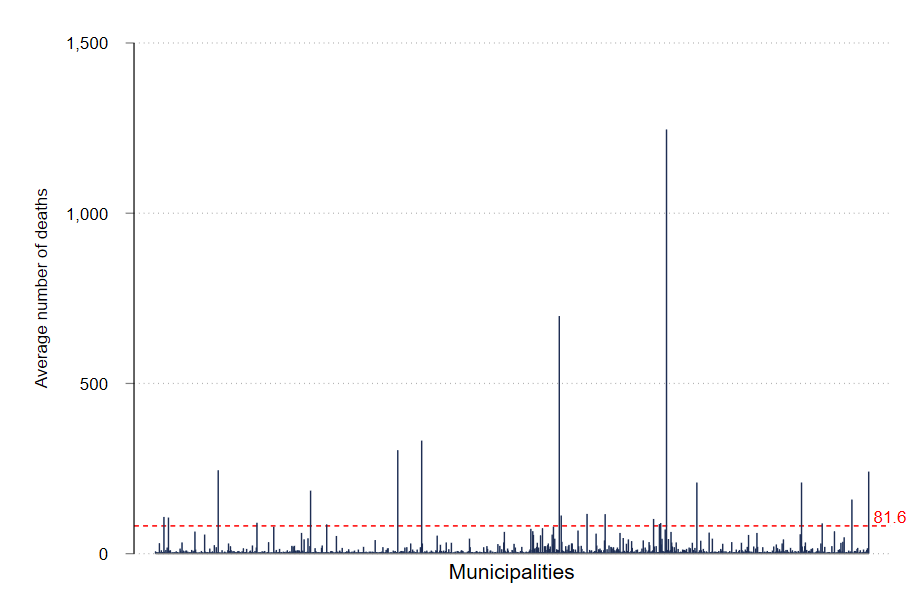


**FIGURE S2** – Average number of deaths across the 1,105 Brazilian municipalities included in the study from 2016 to 2018.

*Note:* The red line represents the overall average number of deaths.


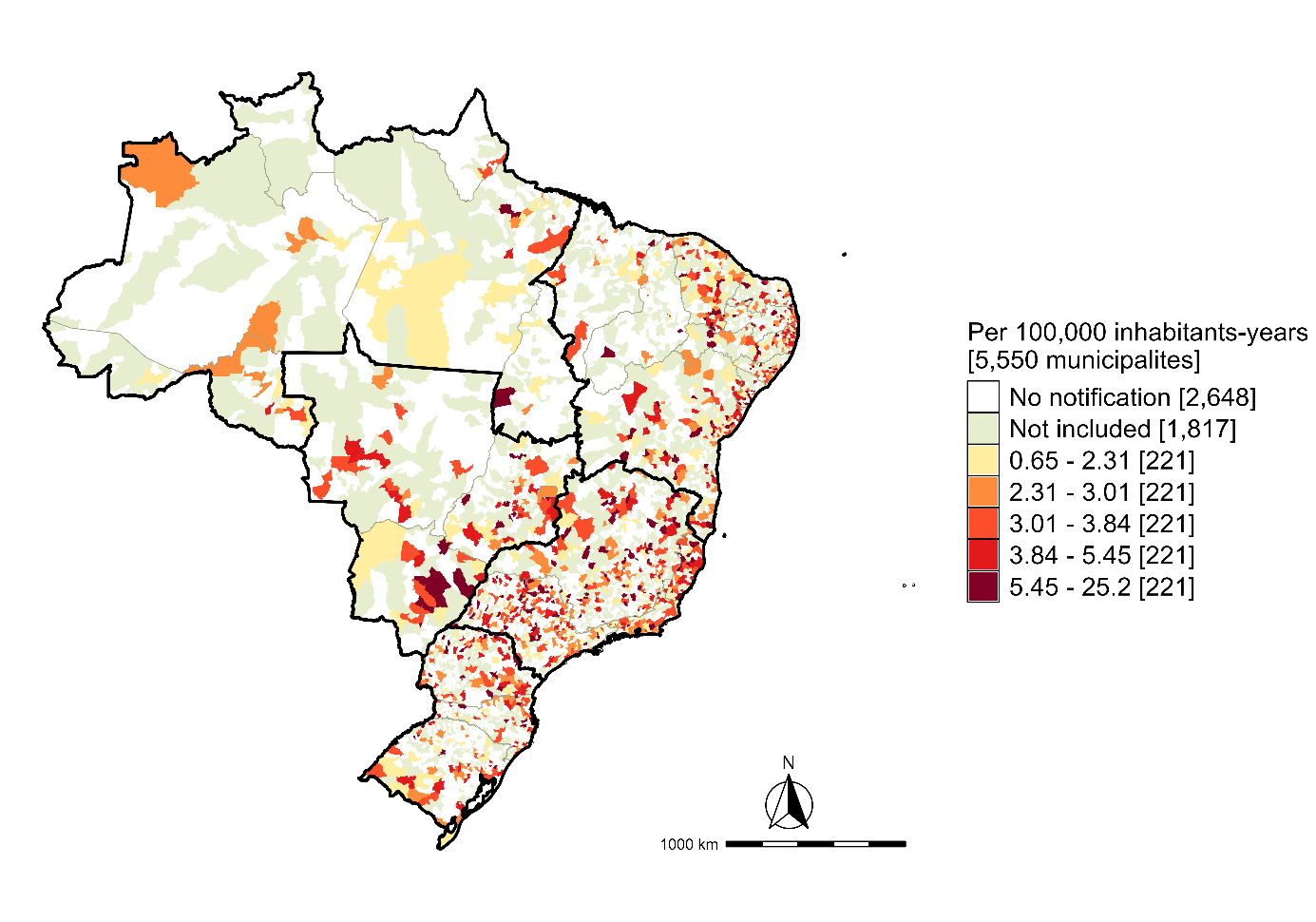
**FIGURE S3**– Age- and sex-standardized oral cancer mortality rate in 1,105 municipalities in Brazil from 2016 to 2018.


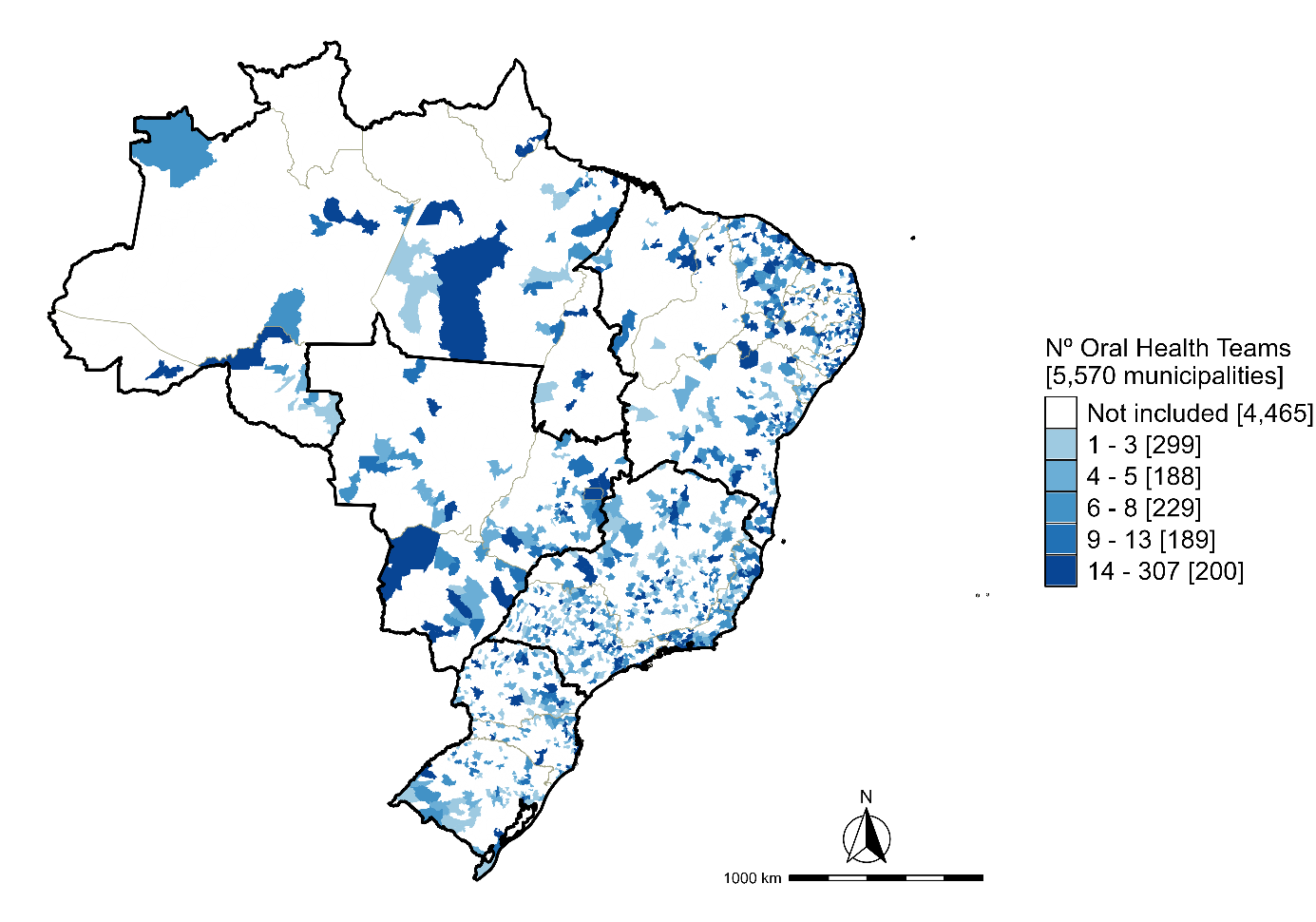


**FIGURE S4** –Distribution of the number of oral health teams in Brazilian municipalities participating in the PMAQ-AB included in the study.


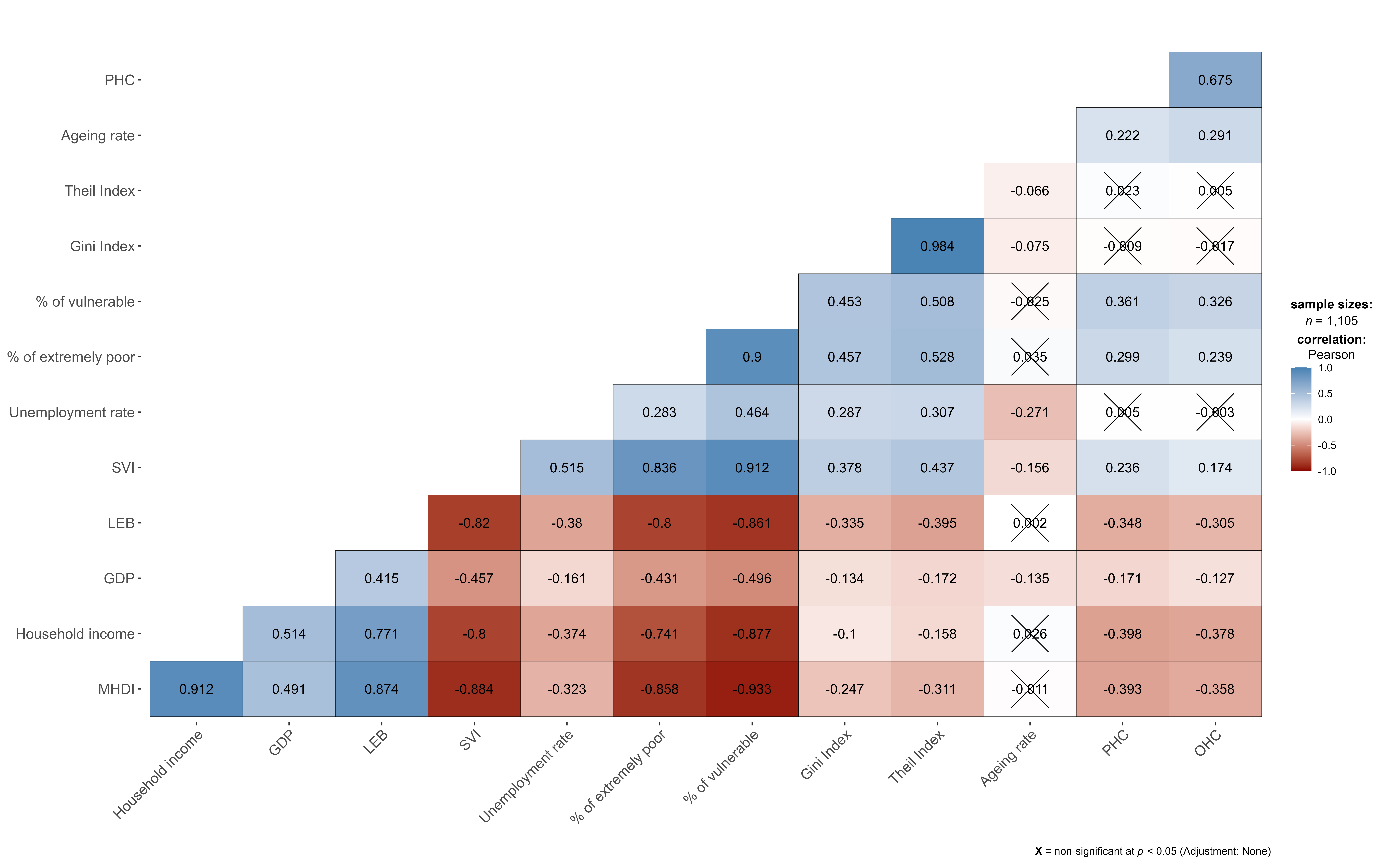


**FIGURE S5 –** Correlation matrix of contextual variables.

*Abbreviations:* GDP: gross domestic product; LEB, life expectancy at birth; MHDI, municipal human development index; OHC, oral health coverage; PHC, primary healthcare; SVI, social vulnerability index.


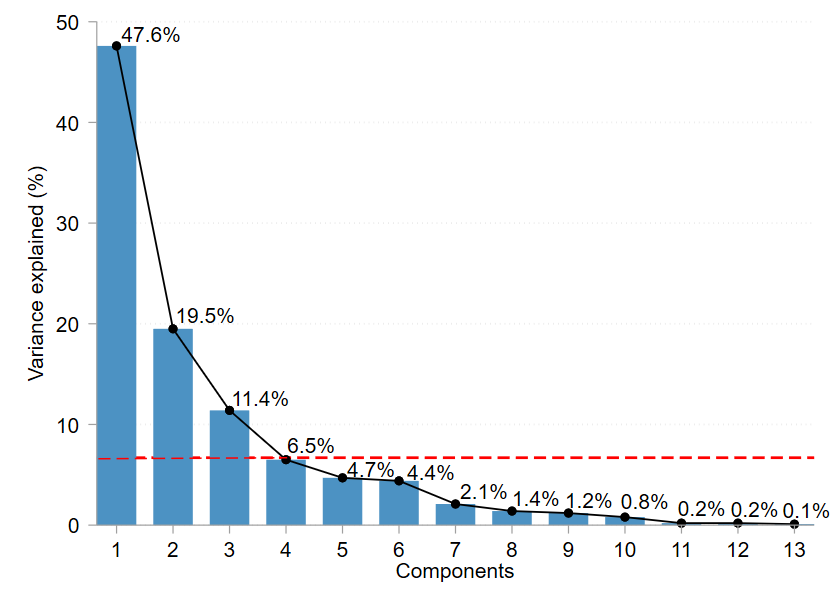


**FIGURE S6 –** Scree plot depicting the percentage of variance explained by each PCA component.

*Note:* The red line differentiates the three principal components with eigenvalue >1.


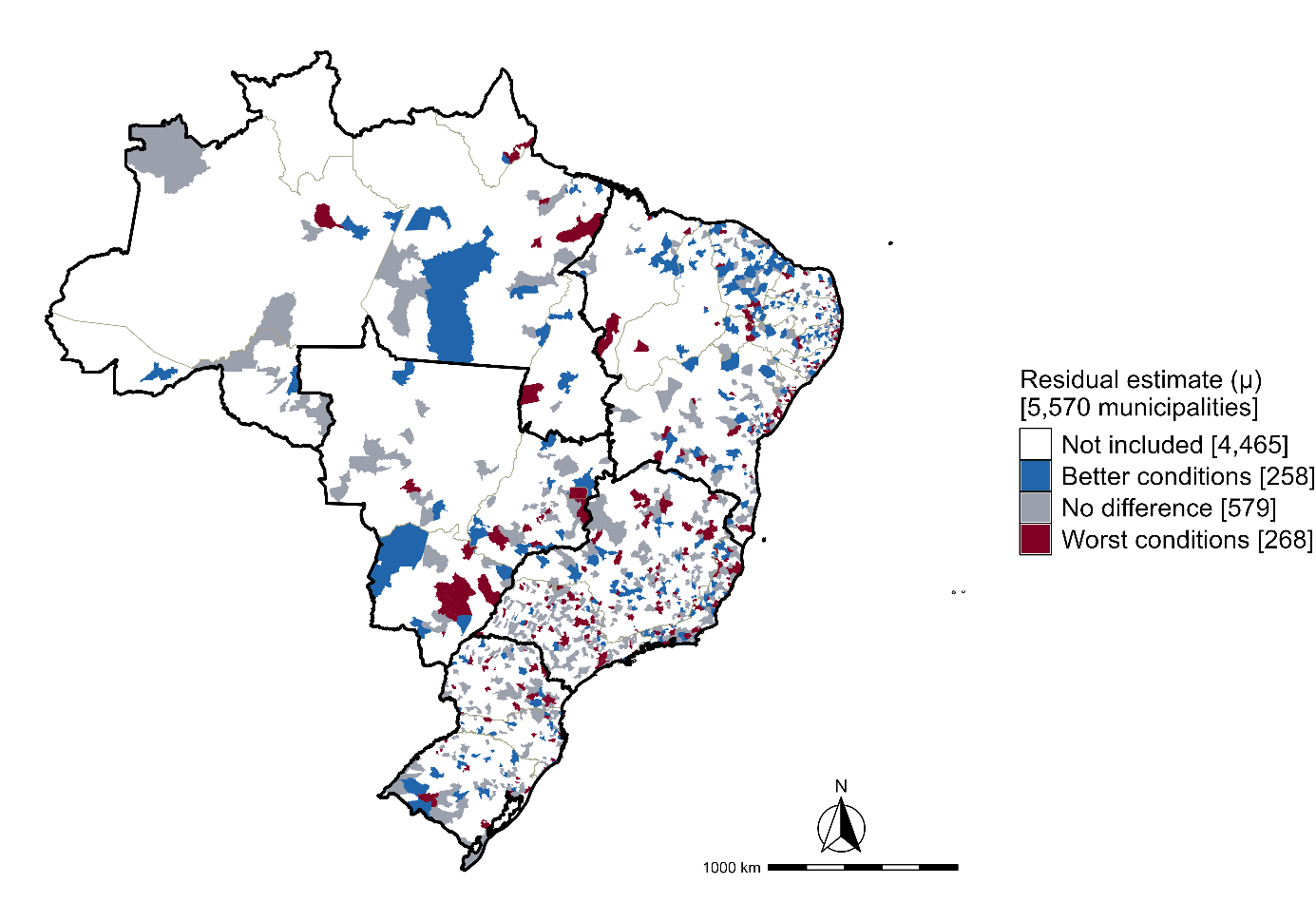


**FIGURE S7** – Effect of Brazilian municipalities on oral cancer mortality rates according to predicted values from the multilevel Poisson regression model.

**REFERENCES**

1. Programa das Nações Unidas para o Desenvolvimento. Atlas do desenvolvimento humano no Brasil [Internet]. [cited 2024 Aug 4]. Available from: http://www.atlasbrasil.org.br/acervo/biblioteca

2. Instituto Brasileiro de Geografia e Estatística. Censo demográfico 2010 [Internet]. [cited 2024 Aug 4]. Available from: https://www.ibge.gov.br

3. Ministério da Saúde do Brasil, Secretaria de Atenção Primária à Saúde. e-Gestor Atenção Básica - Relatórios Públicos [Internet]. [cited 2024 Aug 4]. Available from: https://egestorab.saude.gov.br/

4. Ministério da Saúde do Brasil. Programa Nacional de Melhoria do Acesso e da Qualidade da Atenção Básica (PMAQ-AB): 3^o^ ciclo [Internet]. [cited 2024 Aug 4]. Available from: https://www.gov.br/saude/pt-br/composicao/saps/pmaq
